# Supplementary material for: Encapsulation of Photothermal Nanoparticles in Stealth and pH-Responsive Micelles for Eradication of Infectious Biofilms In Vitro and In Vivo
Source: Nanomaterials (Basel). 2021 Nov 24;11(12):3180. doi: 10.3390/nano11123180 (PMC8706488; doi:10.3390/nano11123180)
Supplement: Supplementary file 1 [file nanomaterials-11-03180-s001.zip › nanomaterials-1450076-supplementary.pdf]

Supplementary Material

Encapsulation of Photothermal Nanoparticles in Stealth and pH-Responsive Micelles for Eradication of Infectious Biofilms In Vitro and In Vivo

Ruifang Gao [1,2,†], Linzhu Su [2,3,†], Tianrong Yu [2,4,†], Jian Liu [4], Henny C. van der Mei [2], Yijin Ren [5], Gaojian Chen [1,*], Linqi Shi [3,*], Brandon W. Peterson [2] and Henk J. Busscher [2,*]

1 College of Chemistry, Chemical Engineering and Materials Science, Soochow University, Suzhou 215006, China; r.gao@umcg.nl
2 University of Groningen, University Medical Center Groningen, Department of Biomedical Engineering 9713 AV Groningen, The Netherlands; sulinzhu@mail.nankai.edu.cn (L.S.); Tianrongyu@yeah.net (T.Y.); h.c.van.der.mei@umcg.nl (H.C.v.d.M.); b.w.peterson@umcg.nl (B.W.P.);
3 Key Laboratory of Functional Polymer Materials of Ministry of Education State, Key Laboratory of Medicinal Chemical Biology, Institute of Polymer Chemistry College of Chemistry, Nankai University, Tianjin 300071, China
4 Jiangsu Key Laboratory for Carbon-Based Functional Materials & Devices, Institute of Functional Nano & Soft Materials (FUNSOM), Soochow University, Suzhou 215006, China; jliu@suda.edu.cn (J.L.)
5 University of Groningen, University Medical Center Groningen, Department of Orthodontics, 9713 AV Groningen, The Netherlands; y.ren@umcg.nl
* Correspondence: gchen@suda.edu.cn (G.C.); shilinqi@nankai.edu.cn (L.S.); h.j.busscher@umcg.nl (H.J.B.)
† These authors contributed equally to this article.

Synthesis of polydopamine-nanoparticles (PDA-NPs)

Synthesis was based on our previous work [1]. In essence, PDA-NPs were prepared through mixing of an aqueous ammonia solution (3 mL, NH_4OH, 28-30%) with absolute ethanol (40 mL) and deionized water (90 mL) under mild stirring at 30ºC for 30 min. Then, 10 mL of dissolved 3-hydroxytyramine hydrochloride (40 mg/mL) was injected at a rate of 25 mL/h, yielding a mixed solution that became brown as the reaction continued. Reaction was conducted for 24 h to form a melanin-like structure. The resulting black product was precipitated with absolute ethanol and collected by centrifugation (11000 g, 5 min). The resulting PDA-NPs were resuspended in deionized water for the dialysis (Mw = 3500 Da, 24 h) against deionized water.

Supplementary Figures

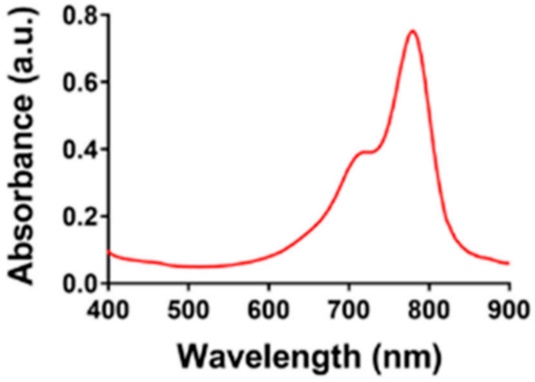

Figure S1. UV–vis absorption spectrum of Indocyanine green (ICG).

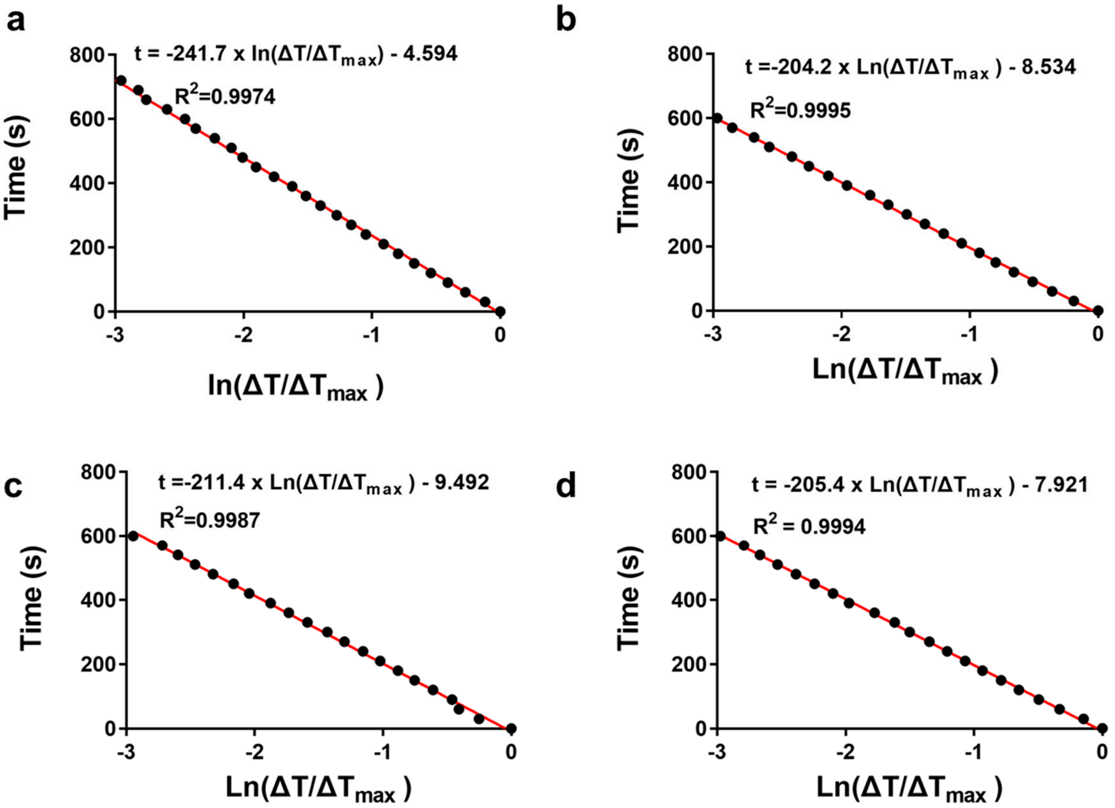

Figure S2. Temperature change expressed as ln(ΔT/ΔTmax) as a function of time during cooling of the system in absence of NIR-irradiation. **(a)** PDA-NPs **(b)** PDA-ICG-NP **(c)** PDA-ICG/PEG micelles **(d)** PDA-ICG/PEG-PAE micelles.

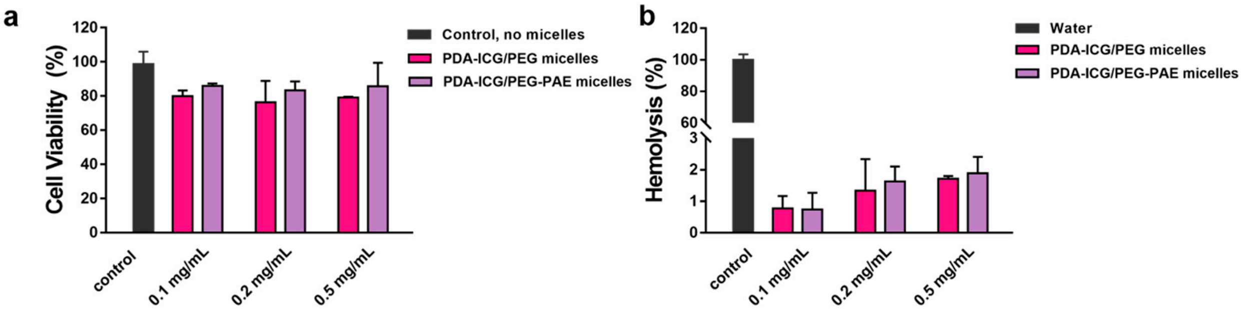

Figure S3. Cytotoxicity and hemolysis of photothermal nanoparticles after micellar encapsulation. **(a)** Viability of L929 fibroblasts after 24 h incubation in presence of different concentrations of PDA-ICG after micellar encapsulation. Viability of fibroblasts incubated in medium only was set at 100%. Data are expressed as means ± standard deviations over triplicate experiments. **(b)** Hemolysis of mouse red blood cells after 3 h exposure to different concentrations of PDA-ICG after micellar encapsulation. Hemoglobin absorption of cells exposed to water was set at 100%. Data are expressed as means ± standard deviations over three separate experiments (panel a) or over three mice in each group (panel b). No statistically significant differences were observed.

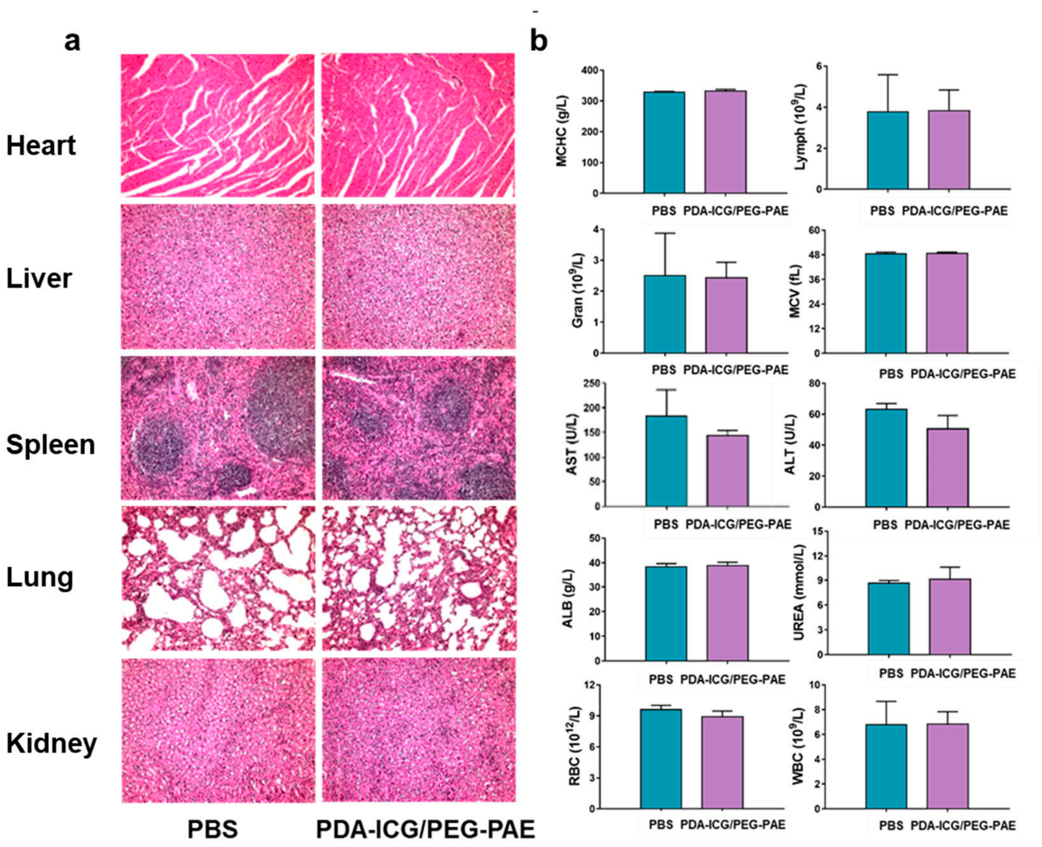

Figure S4. *In vivo* biosafety of photothermal nanoparticles after micellar encapsulation. PDA-ICG/PEG-PAE micelles suspended in PBS or PBS (200 μL) were injected daily into the tail vein of a mouse over 3 days consecutive days. Organ tissues and blood were collected 14 days after arresting injection. **(a)** Histological images of internal organ tissues after hematoxylin and eosin (H&E) staining. **(b)** Biochemical blood parameters. MCHC stands for mean corpuscular hemoglobin concentration, Gran for granulocytes, AST for aspartate transferase, ALB for albumin and RBC for white and red blood cell, Lymph for lymphocytes, MCV for mean cell volume, ALT for alanine transferase, UREA for urea nitrogen and WBC for levels of white blood cells, respectively. Data represent means with standard deviations over 3 mice in each group. No statistically significant differences were observed.

References

1. Gao, R.; Van der Mei, H.C.; Ren, Y.; Chen, H.; Chen, G.; Busscher, H.J.; Peterson, B.W. Thermo-Resistance of Eskape-Panel Pathogens, Eradication and Growth Prevention of an Infectious Biofilm by Photothermal, Polydopamine-Nanoparticles *In Vitro*. *Nanomedicine* **2021**, *32*, 102324, doi: 10.1016/j.nano.2020.102324
